# Supplementation of Mixed Organic Acids Improves Growth Performance, Meat Quality, Gut Morphology and Volatile Fatty Acids of Broiler Chicken

**DOI:** 10.3390/ani11113020

**Published:** 2021-10-20

**Authors:** Jiayu Ma, Jian Wang, Shad Mahfuz, Shenfei Long, Di Wu, Jie Gao, Xiangshu Piao

**Affiliations:** 1 State Key Laboratory of Animal Nutrition, College of Animal Science and Technology, China Agricultural University, Beijing 100193, China; jiayuma@cau.edu.cn (J.M.); S20173040498@cau.edu.cn (J.W.); shadmahfuz@yahoo.com (S.M.); longshenfei@cau.edu.cn (S.L.); bs20213040377@cau.edu.cn (D.W.); gaojie01@caas.cn (J.G.); 2 Department of Animal Nutrition, Faculty of Veterinary, Animal and Biomedical Sciences, Sylhet Agricultural University, Sylhet 3100, Bangladesh; 3 Institute of Animal Sciences, Chinese Academy of Agricultural Sciences, Beijing 100193, China

**Keywords:** mixed organic acid, broilers, growth performance, meat quality, gut morphology

## Abstract

**Simple Summary:**

Organic acid as a green feed additive is increasingly favoured by enterprises and scholars, but little emphasis has been placed on the effect of organic acids on broiler meat quality and lipid profile. Therefore, this study observed that mixed organic acids improve broiler growth performance, meat quality as well as muscle lipid profile, which suggests that mixed organic acids can be an effective measure to prevent meat quality decline in chicken meat.

**Abstract:**

**Background**: Organic acid as a green feed additive is increasingly favoured by enterprises and scholars, but little emphasis has been placed on the effect of organic acids on broiler meat quality. **Methods**: A total of 192 male chicks (one-day-old, weighted 48.40 ± 0.64 g) were selected to investigate the effect of mixed organic acids (MOA) on growth performance, meat quality as well as fatty acids profile. Chicks were randomly allocated to three treatments with eight replicates and eight chicks per replicate, including a corn–soybean basal diet with 0 (CON), 3000 mg/kg (low MOA; LMOA), and 6000 mg/kg (high MOA; HMOA) MOA. The experiment was divided into starter (d 1–d 21) and grower (d 22–d 42) phases. Results: Broilers supplemented with LMOA and HMOA enhanced (*p* < 0.05) the final body weight and average daily gain in the grower and overall phases. An improved (*p* < 0.05) feed conversion ratio in the grower and overall phases was observed in broilers supplemented with LMOA. The breast and thigh muscles pH24h were higher (*p* < 0.05) in broilers fed with HMOA and the redness in thigh meat was also improved (*p* < 0.05). Additionally, supplementing LMOA increased (*p* < 0.05) the saturated fatty acids, unsaturated fatty acids and the ratio of polyunsaturated fatty acids to saturated fatty acids in breast meat. A positive effect occurred (*p* < 0.05) on jejunal villus height and ileal crypt depth in 21 d broilers supplemented with HMOA. **Conclusion**: Our findings indicated that dietary supplementation of MOA could improve the growth performance, meat quality, and fatty acids profile, as well as intestinal morphology. Furthermore, diets supplemented with mixed organic acids at 3000 mg/kg may be more desirable, considering the overall experimental results in broilers.

## 1. Introduction

With the growing awareness of antibiotic abuse and its side-effects (drug resistance, residual, toxicity and environmental pollution), as well as considerations based on food safety and human health in several countries [1] such as the European Union [2], the United States [3], China [4], and India, have issued regulations to ban and restrict the use of antibiotics [5], which has undoubtedly brought tremendous challenges for the development of feed enterprises. Therefore, the exploration of green, safe and pollution-free antibiotic alternatives is an inevitable trend in the development of animal husbandry. With the continuous attempts and investigations of scholars on antibiotic alternative products for feeding, a series of green additives for improving the growth performance and intestinal health of livestock and poultry are attracting the attention of the feed industry, such as plant extracts, yeast, minerals, antimicrobial peptides and probiotics [6]. Organic acid, as a green feed additive, has been preferred by enterprises because of its three-free characteristics (pollution-free, drug resistance-free and residual-free) [7]. With in-depth research, scholars have discovered that organic acids are capable of improving growth performance [8,9,10,11], increasing the immune and antioxidant properties of livestock [12], improving intestinal function [13,14,15], and regulating their intestinal microbiota [16,17]—especially effective on weaning pigs [14,18]. However, numerous studies have mainly focused on the performance and intestinal health of animals. Limited literature understands the effects of organic acids on meat quality characteristics and the fatty acids profile of livestock—especially broilers. Moreover, the findings of several scholars concerning the improvement of broiler intestinal morphology by dietary organic acids are inconsistent [19,20]. Thus, the objective of this work was to investigate the effect of dietary supplementation with high and low concentrations of mixed organic acids (MOA) on performance, intestinal morphology and volatile fatty acids of broilers. The major concern was whether the variation in meat colour, pH, and lipid profile of the breast and thigh meat was dependent on the dose effect of MOA.

## 2. Materials and Methods

The research was conducted in an experimental chicken house at the China Agricultural University. The procedures of this research were sanctioned by the Institutional Animal Care and Use Committee of China Agricultural University (Beijing, China, AW09089104-1).

### 2.1. Mixed Organic Acids Products

The MOA product (named Fysal MP, code: 2017SZ34) employed in this research was offered by Nutreco (Amsterdam, The Netherlands). The MOA product mainly consists of 11% formic acid, 13% ammonium formate, 5.1% acetic acid, 10% propionic acid, 4.2% lactic acid, and other lower levels (≤2%) of organic acids (sorbic acid and citric acid). The carrier is silica.

### 2.2. Experimental Design and Feed Management

A total of 192 one-day-old male chicks, weighing 48.40 ± 0.64 g, were purchased from a commercial hatchery (Arbor Acres Poultry Farm, Beijing, China) and transported to the experimental poultry house by truck within 30 min while the temperature was maintained at 35 °C. The one-day-old male chicks were weighed upon arrival and tagged with wing rings. The chicks were randomized into three treatments with eight replicates per treatment and eight broilers per replicate. The dietary supplementation of MOA at 0 mg/kg (CON), 3000 mg/kg (low MOA, LMOA) and 6000 mg/kg (high MOA, HMOA) of corn-soybean meal basal diets were implemented, respectively. The experiment was divided into two phases: starter (d 1 to d 21) and grower (d 22 to d 42). The diet composition and nutrient levels (Table 1) were designed in accordance with Ma et al. [17], which also satisfied or exceeded the nutrient requirements of Arbor Acres broiler requirements [21].

The experimental poultry house was equipped with forced ventilation and radiant heating, and the experiment was performed using a three-level cage approach. The initial temperature of the room was controlled at 35 °C and then lowered by 2–3 °C weekly until it reached 22 °C, with relative humidity maintained at 45–55%. However, the temperature and humidity should be slightly adjusted in accordance with the growth and health conditions of broilers under current management. Additionally, a 23 h light: 1 h dark light program was implemented in the room and the immunization procedures were strictly in accordance with the commercial broiler feeding management system. All chicks were vaccinated with Newcastle disease and infectious bronchitis combined live vaccine (Lasota+H120, supplied by Qilu Animal Health Products Co., Ltd. Shandong, China) on the 7th day of the experiment by injection and drinking water. Immunization with infectious bursal weak vaccine was conducted on the 14th day of the experiment. The broilers were allowed to feed intake (diets fed in the form of mash) and drink freely overall, the room temperature, humidity, light and broiler growth were noted daily, and mortality and culling rates were recorded in detail. Feed was recorded every day and broilers were weighed on the 21st and 42nd day of experiment, and average daily gain (ADG), average daily feed intake (ADFI) and feed conversion ratio (FCR) were recorded and calculated for 1–21 d, 22–42 d and 1–42 d. No broiler mortality occurred during the experiment.

### 2.3. Sampling Procedure

The experiment continued for a total of 42 days. On the 21st day and 42nd day of the experiment, a broiler chicken with the mean body weight was selected from each replicate, sacrificed by the cervical dislocation method, and the abdominal cavity immediately dissected. Approximately 1–2 cm of intestinal samples were collected from the duodenum, jejunum and ileum at 1/3 of the posterior segment, respectively. The intestinal chyme was washed off with saline (0.9% NaCl) before being fixed immediately in a prepared 4% paraformaldehyde solution for intestinal morphological examination of the 21st and 42nd day’s samples of broiler chickens, respectively. Then, the chyme was sampled from the mid cecum of broilers (sampled after the knot on both sides) and collected into sterile 5 mL centrifuge tubes, before being quickly placed in liquid nitrogen and stored in a refrigerator at −80 °C for the determination of the 21st and 42nd day’s cecal volatile fatty acids content of broiler chickens, respectively. Additionally, approximately 30 g of the right breast muscles and right thigh muscles were collected on the 42nd day of the experiment for detection of the 42nd day’s meat quality and fatty acids profile of broiler chickens. Throughout the collection process, all samples were sampled in duplicate (one for analysis, one for backup).

### 2.4. Meat Quality

The right pectoralis muscle and the right thigh muscle were taken at the time of slaughter, and the probe was inserted into the meat using a calibrated portable pH meter (OPTP-STAR, Metz, Germany) and determined at three different positions for each piece of meat. The mean value was calculated and recorded as pH_45 min_. Meanwhile, the lightness (L*), redness (a*), and yellowness (b*) of the muscle were measured three times with a Medan colorimeter (CR-410, Konica Minolta Holdings, Tokyo, Japan) and the mean value computed. Then, the right breast and thigh were stored at 4 °C until 24 h and the pH value, L*, a*, and b* were measured again three times at three different positions. The mean value was calculated, recorded as pH_24 h_ and the ΔpH calculated (ΔpH = pH_45 min_ − pH_24 h_) according to Long et al. [22].

Additionally, the middle part of the left pectoralis muscle and the left thigh was taken, trimmed to length (30 mm) × width (15 mm) × thickness (5 mm), weighed, then caught by a wire at one end—making sure the muscle fibers were placed vertically upward—put into an airy plastic bag without the sample touching the bag wall, and tied and hung in the refrigerator at 4 °C. After 24 h, the meat samples were dried with filter paper, and weighed to calculate the drip loss based on the percentage of weight before and after hanging.

### 2.5. Intestinal Morphometry

After 48 h of fixation in 4% paraformaldehyde solution, the sections of small intestinal samples (duodenum, jejunum and ileum) were washed, excised and dehydrated with ethanol for 24 h, embedded in paraffin, and cut into four cross-sections and stained with hematoxylin-eosin. Finally, samples were morphologically tested by light microscopy (Olympus CX31, Tokyo, Japan), and ten intact, well-oriented villous-crypt units measured in each section. The villus height (VH) was estimated from the top of the villus to the junction of villus crypts; the crypt depth (CD) was measured as the depth of villus invagination and calculated the ratio of villus height to crypt depth (VH/CD).

### 2.6. Muscle Fatty Acids Profile

Approximately 20 g of breast and thigh muscle samples were lyophilized for 60 h by lyophilizer and thawed to determine the muscle fatty acids concentrations of the 42nd day broiler breast and thigh muscle samples. Lipid-source fatty acids profiles were quantified by gas chromatography (6890N, Agilent Technologies, Wilmington, USA) according to the methodology of Sukhija and Palmquist [23], with minor amendments. The specific steps are as follows: First approximately 0.2 g sample was weighed in the hydrolysis tube, 4 mL chloroacetylmethanol solution (10%) and 1 mL internal standard solution (1.0 mg/mL, undecanoic acid) added, supplemented with 1 mL of n-hexane, and the bottle capped tightly and placed in a thermostat water bath cauldron at 80 °C for 2 h. After cooling, 5 mL of potassium carbonate (7%) was added, shaken homogeneously and centrifuged at 1000 r/min for 5 min, finally filtered through a 0.2 μm membrane and loaded into the injection vial, and detected on the machine. The conditions of gas chromatography (single ramp, gradient heating mode) were as follows: FID detector, column DB-23 quartz capillary column (60.0 m × 250 μm × 0.25 μm); Inlet temperature was 260 °C, injection volume was 1 μL (internal standard:), the splitting ratio was 30:1, and carrier gas (helium, 99.99%) flow rate was 2 mL/min. Procedural ramp-up was used: 50 °C for 2 min, 10 °C /min to 170 °C and kept for 10 min, 4 °C /min to 180 °C and maintained for 10 min, 2 °C /min to 220 °C and maintained for 10 min. The detector temperature was 270 °C, hydrogen, 40 mL/min, airflow, 400 mL/min, tail blow flow (helium), 40 mL/min. The duration of analysis was 50 min per sample. Furthermore, the fatty acids composition of breast and thigh muscles was computed based on g/100 g of total fatty acids in the tissues (dry matter-basis).

### 2.7. Volatile Fatty Acids

The cecum chyme was removed from the −80 °C refrigerator, and approximately 0.5 g of the chyme sample was weighed and placed in a 10 mL polypropylene tube, supplemented with 8 mL of deionized water, and centrifuged at 15000 rpm for 10 min at 4 °C after 30 min of ultrasonic shock. The supernatant was extracted and diluted tenfold, then filtered through a 0.22 μm filter. The 25 μL filtrate was transferred into the sample bottle of a gas chromatograph; the concentrations of lactic acid and volatile fatty acids were determined high-performance ion chromatography with an ICS-3000 (Dionex, Sunnyvale, CA, USA) and determined by conductivity detection. The external standard solution containing eight acids (lactic acid, acetic acid, propionic acid, formic acid, isobutyric acid, butyric acid, isovaleric acid and valeric acid) were purchased from Sigma-Aldrich (Sigma-Aldrich, Saint Louis, MO, USA). The organic acids were separated on an AS11 analytical column (250 mm× 4 mm) and an AG11 guard column under the following gradient conditions: gradient was carried out with potassium hydroxide; 0–5 min, 0.8–1.5 mM; 5–10 min, 1.5–2.5 mM, 10–15 min, 2.5 mM; the flow rate was 1.0 mL/min.

### 2.8. Statistical Analysis

All indicators were calculated on an individual basis, with the exception of growth performance data, which was calculated based on cages. The raw data was preliminarily organized by Excel software (Microsoft, Redmond, WA, USA). The experimental data were analysed by one way-ANOVA analysis using the GLM program and Duncan’s multiple comparison was conducted to analyse the differences between treatments using SAS 9.2 (SAS Institute, Cary, NC, USA) software in a completely randomized design. The linear and quadratic comparisons were applied to determine the dose-effect of MOA in broiler chickens. Statistical significance was considered when *p* ≤ 0.05 and a trend with significant differences was regarded when 0.05 < *p* ≤ 0.10.

## 3. Results

### 3.1. Growth Performance

In the grower period (Table 2), compared with the control group, the diets supplemented with LMOA and HMOA increased (*p <* 0.05) the final weight by 11.11% and 8.0%, enhanced (*p <* 0.05) by 11.9% and 9.9% in ADG, and improved (*p <* 0.05) by 9.3% and 8.2% in FCR, respectively. In the overall phase, an enhanced (*p <* 0.05) ADG of broilers supplemented with LMOA and HMOA was noticed, which increased by 11.4% and 8.3%, respectively. Additionally, an improved (*p <* 0.05) FCR of broilers supplemented with LMOA was observed, which improved by 8.6%.

### 3.2. Meat Quality

In breast meat (Table 3), a higher (*p <* 0.05) pH at 24 h and a lower (*p* < 0.05) ΔpH were noticed in 42 d broilers supplemented with HMOA. Additionally, a linearly decreased trend (*p* = 0.09) of luminosity at 45 min and a linearly increased (*p* = 0.06) trend of redness at 24 h was observed in broilers supplemented with MOA. In thigh meat, a linearly enhanced (*p* < 0.05) pH and redness at 24 h were noted in 42 d broilers supplemented with HMOA. Additionally, a linearly decreased trend (*p* = 0.09) of ΔpH was observed in broilers fed MOA.

### 3.3. Lipid Profile

In breast meat (Table 4), a quadratically lower content (*p* ≤ 0.05) of palmitic acid (C16:0) and saturated fatty acids was observed in broilers supplemented with LMOA. Additionally, broilers fed with LMOA quadratically increased (*p <* 0.05) the concentration of α-linolenic acid (C18:3n3) and unsaturated fatty acids (UFAs), as well as the ratio of polyunsaturated fatty acids to saturated fatty acids (P/S). In thigh meat (Table 5), a linearly lower content (*p <* 0.05) of docosanoic acid (C22:0) and tricosanoicacid (C23:0) was noticed in broilers with dietary HMOA supplementation. Moreover, dietary LMOA supplementation quadratically enhanced (*p <* 0.05) the concentration of α-linolenic acid (C18:3n3) and linoleic acid (C18:2n6c), as well as the ratio of polyunsaturated fatty acids to saturated fatty acids (P/S).

### 3.4. Intestinal Morphology

In the starter period (Table 6), dietary HMOA supplementation linearly declined (*p <* 0.05) the crypt depth in the duodenum and ileum. Additionally, a linearly enhanced (*p <* 0.05) villus height in the jejunum and the ratio of villus height to crypt depth in the ileum was noticed in the HMOA group. In the grower period (Table 7), diet supplemented with LMOA linearly increased (*p <* 0.05) the villus height in the duodenum and ileum, respectively. The HMOA group linearly enhanced (*p <* 0.05) the duodenal villus height. However, diet supplemented with LMOA increased (*p <* 0.05) the jejunal crypt depth, and in the HMOA group, a linearly enhanced (*p <* 0.05) duodenal crypt depth was noticed.

### 3.5. Volatile Fatty Acids in Cecum

In the starter period (Table 8), diet supplemented with LMOA and HMOA increased (*p <* 0.05) the content of formate in the cecum. However, no significant difference was observed among treatments with the exception of formate. In the grower period, a higher (*p <* 0.05) concentration of acetate, propionate, butyrate and isobutyrate was noticed in the LMOA group. However, no significant difference between the control group and HMOA group was noted.

## 4. Discussion

The key role of organic acids in livestock and poultry production has been reviewed previously [1,6,24]. However, all studies focused on the effects of organic acids on growth performance [15,25], immuno-antioxidant capacity (challenged model) [20,26], intestinal function [27,28] and intestinal microbiota [29] of poultry. The majority of the results are variable owing to inconsistencies in the experimental environment as well as the composition and concentration of MOA. But there is a certainty that dietary supplementation with MOA is beneficial to broiler performance and health. Such positive effects may be attributed to a reduction in buffering capacity and improvement in nutrient digestibility, as well as a lowering of pH values in the feed and gastrointestinal tract, which performs as a barrier resistant to pathogenic microorganisms susceptible to low pH value [30]. In our findings, diet supplemented with MOA could enhance the final weight and improve the ADG as well as the FCR of broilers in the grower and overall phases, but no significant difference was observed in the starter period—which indicated that the effect of MOA is growth phase-dependent and dominated in the grower period. The explanation probably lies in the fact that the chicks in the starter period are developing and unable to effectively absorb and use the ingredients contained in the MOA to improve nutrient digestibility and growth performance, while as the body grows, the intestinal function tends to be perfected and the digestibility and absorption of various nutrients are enhanced, thus improving broiler growth performance. In addition, the effects of dietary addition of LMOA and HMOA on broiler growth performance appeared to be the same, but superior to FCR in the grower period and overall—which was similar to Panda et al. [31], who showed that dietary supplementation with 0.4% organic acid improved the ADG and FCR, but no supplementary advantage on these parameters was noticed while increasing the content of organic acid from 0.4% to 0.6% in the diets. Therefore, broiler diets supplementation with LMOA is preferable to HMOA in terms of management cost.

Meat with lower water retention capacity is prone to rapid loss of nutrients and minerals [32]. The changes in pH are associated with glycolysis and directly affect the meat quality of broilers, which is one of the key nutritional indicators reflecting the nutritional quality of chicken meat after slaughter [33]. Additionally, the rapid decrease in pH value triggered the denaturation of a large amount of protein and fat in cold contracted muscle. The increase in protein–fat denaturation could reduce the solubility of the protein in muscle and the content and concentration of melanin in muscle, which have serious impacts on the colour and flavour of the muscle [34]. In our experiment, diet supplemented with HMOA improved the pH at 24 h of breast and thigh muscles, which is probably attributed to the fact that the components of organic acids (acetic acid, propionic acid, mainly butyric acid) supply energy to chicken tissues and avoid accelerated glycolysis in chicken tissues, resulting in a lower concentration of lactic acid in chicken meat and a slower decrease in chicken pH after slaughter. Additionally, the study of Galli et al. [8] revealed that diet supplemented with microencapsulated organic acids (formic, phosphoric, lactic, acetic, butyric and propionic acids) could improve the antioxidant status of meat. Therefore, it may be associated with improved antioxidant properties in the muscle, which alleviated muscle stress, reduced the rate of glycolysis, decreased lactate levels, and lowered the rate of pH decline.

Meat colour L* values (luminosity) illustrate the amount of oxygenated myoglobin, a * values (redness) indicate the level and presence of deoxygenated myoglobin in the muscle, and b * values (yellowness) reflect the content of myoglobin that has been oxidized to high iron myoglobin; thus, more redness is desirable in chicken meat [8,35]. In general, in red meat, placement in the outer packaging resulted in the oxidation of oxymyoglobin into methemoglobin with lower red values, which are relevant to the antioxidant properties of chicken [36] ΔpH for evaluating the speed of decline in muscle pH values [22]. Our findings showed that diet supplemented with HMOA could reduce the ΔpH in the breast muscle and that a higher redness was observed in the thigh muscle. This may be linked to the improvement of the antioxidant status of the muscles by dietary supplementation with MOA, which avoids the conversion of oxymyoglobin into methemoglobin and thus improves redness [36]. Additionally, our results indicated that a slower reduction in pH (improvement of ΔpH) could prevent deformation of proteins and fats in the muscle, thereby facilitating protein solubility and increasing the amount of melanin in the muscle to improve meat quality [34]. However, Nguyen et al. [37] supplied 20, 30, 40, 50, 60 mg/kg of MOA (fumaric acid, citric acid and malic acid) to the diets of broilers, respectively, but there was no difference between control and treatments on meat quality in broilers, which may be correlated with the concentration of MOA supplemented in the diet. Moreover, one study demonstrated that the umami of chicken meat was strongest when the pH value was approximately 6.0, that the umami of chicken meat was lost when the pH value was greater than 7.0, and that the pH value of thigh muscle was on average 0.3–0.5 units higher than that of breast muscle according to the breed [38]—which was also consistent with our findings. Therefore, dietary addition of HMOA improved the muscle quality of broilers by modifying muscle pH, slowing the rate of muscle pH decline and regulating meat colour.

The fatty acid content of chicken, especially unsaturated fatty acids, serves as an essential precursor to the flavour of chicken meat, such as linoleic acid, linolenic acid and arachidonic acid [39]. Studies have indicated that the degradation products (hydroperoxide) produced by the oxidative decomposition of unsaturated fatty acids (UFA) and their participation in the Maillard reaction produce not only fatty aromas but also characteristic flavours of meat, such as alcohols, aldehydes, ketones, furans, and other volatile compounds that contribute to the flavour of chicken meat [40]. Additionally, according to the literature, UFAs could reduce the incidence of human cardiovascular diseases [41] Thus, efforts to decrease the content of SFAs and enhance the content of UFAs are anticipated. In our findings, an increased level of UFAs and a reduced level of SFAs as well as an improved ratio of PUFAs to SFAs in the meat of broilers supplemented with LMOA were observed, which was parallel with Galli et al. [8], who indicated that dietary microencapsulated organic acids lowered the level of SFAs and increased the level of UFAs in broiler meat. This phenomenon probably resulted from the involvement of some components of MOA in fatty acid metabolism in the liver, reducing the process of ab initio synthesis of fatty acids, as well as the increased synthesis of desaturases Δ5, Δ6 [42] and Δ9 [27]. However, PUFA-enriched chicken contains a greater proportion of fatty acids with double bonds, which may influence oxidative stability and increase its susceptibility to oxidation, thus affecting the meat quality of the chicken [43,44]. Moreover, the oxidation process may result in the formation of potentially toxic compounds in relation to meat quality and reduce its shelf life [45]. Therefore, the analysis of the antioxidant status of chicken meat quality is necessary, which is the point of our further focused research. Interestingly, Gebauer et al. [46] noted that the paramount n-3 fatty acid in the human diet is linolenic acid (LNA, C18:3n3)—focusing on LNA since it is a precursor for the synthesis of eicosapentaenoic acid (C20:5n3) and docosahexaenoic acid (C22:6n3). Furthermore, LNA and linoleic acid (LA, C18:2n6) are required to support human hemoglobin synthesis and cell division [47]. Therefore, our studies also reinforce these positive findings, as we observed a higher percentage of LNA and LA in the meat of broilers supplemented with LMOA.

Favourable intestinal morphology indicated improvements in intestinal nutrient digestibility and absorption, with the possibility of further contributing to improved growth performance [48]. In the current study, supplementation of LMOA and HMOA enhanced intestinal morphology, presenting a positive impact on villus height, crypt depth, and the ratio of villus height to crypt depth in the duodenum, jejunum and ileum of 21 d and 42 d broilers, which were similar to previous findings [23,49,50]. This is mainly because MOA could stimulate energy metabolism as a substrate of the tricarboxylic acid cycle, providing energy directly to intestinal epithelial cells, facilitating rapid renewal and proliferation of intestinal epithelial cells, and improving the height of intestinal villi—which could save a lot of time compared with the energy provided by the glycolytic pathway [51,52]. Conversely, Fascina et al. [53] reported that supplementation of organic acids failed to improve the intestinal morphology of broilers, which may be related to the plant-derived additives added to the diet and the lower concentration of organic acids, as well as not encapsulating the organic acids so that they cannot access the intestine to perform their function. Surprisingly, in the present findings, feeding LMOA and HMOA increased the jejunal crypt depth and duodenal crypt depth, respectively, which could reduce the ability of broilers to absorb nutrients. This phenomenon may be caused by the high percentage (5.1%) of acetic acid in MOA products, which temporarily damaged the intestinal mucosa and further induced intestinal inflammation [54,55]. Therefore, our next consideration was to remove the acetic acid portion of the MOA product and further understand whether the damaging effect on the small intestine was due to the high percentage of acetic acid.

Short-chain fatty acids are generated from non-host-digestible carbohydrates by microbial fermentation and are modulated by both the intestinal environment and intestinal microbes. According to the literature, short-chain fatty acids (butyrate), which serve as transmitters to regulate intestinal epithelial barrier function and information exchange between host and microorganisms, have anti-inflammatory and immune-enhancing effects [56,57]. Our results demonstrated that dietary supplementation of MOA enhanced the content of cecal formate in 21 d broilers, which is probably associated with the composition of MOA product (mainly formate). Additionally, an enhanced acetate, butyrate and isobutyrate were noticed in 42 d broilers supplementing LMOA, which is in parallel with Aljumaah et al. [58], who indicated that feeding with MOA increased the level of butyrate, acetate and isobutyrate in the cecum of broilers. This was probably correlated with the proliferation of acid-producing bacteria (such as Acetobacterium balch, Clostridium butyricum) in the posterior intestine after stimulation by the low pH of MOA, which will be verified in our next microbiome work. Moreover, much literature has documented that acetate and butyrate were capable of positively impacting the energy status of the host by activating glyoxylate pathway enzymes that provide a carbon source for the gut microbiota [59,60,61]. Therefore, dietary addition of MOA could improve the health status of broilers by increasing the content of volatile fatty acids in the cecum.

## 5. Conclusions

In summary, a positive impact was observed on growth performance, meat quality and a beneficial change was noticed in the composition of muscle lipid fatty acid and intestinal volatile fatty acid content in broiler diets supplemented with MOA. Our next work would adjust the organic acid ratio (such as lowering or removing the acetic acid) of MOA products, concentrating on the effects of MOA at different levels on the antioxidant status of muscle, intestinal morphology, and the gut microbiome of broilers to confirm our hypothesis. Additionally, considering the effectiveness of MOA in the current study as well as the cost, the LMOA group might be superior compared to the HMOA.

## Figures and Tables

**Table 1:** Composition and nutrient levels of basal diets (DM ^1^ basis).

Ingredients (%)	Phase 1 (d 1 to d 21)	Phase 2 (d 22 to d 42)
Corn, 8.2 % CP ^1^	61.74	65.17
Soybean meal, 46% CP	28.50	24.50
Fish meal, 64.7% CP	3.29	3.33
Soy oil	2.90	3.81
Dicalcium phosphate	1.30	1.20
Limestone	1.28	1.10
Salt	0.30	0.30
L-lysine HCl, 78%	0.00	0.00
DL-Methionine, 98%	0.15	0.05
L-Threonine, 98%	0.04	0.04
Vitamin-mineral premix ^2^	0.50	0.50
Total	100	100
Nutritional levels ^3^		
Metabolizable energy, kcal/kg	3050.73	3150.11
Crude protein	20.68	19.06
Calcium	1.02	0.91
Digestible phosphorus	0.43	0.40
Standardized ileal digestible lysine	0.80	0.72
Standardized ileal digestible methionine	0.27	0.25
Standardized ileal digestible threonine	0.58	0.53

^1^ DM: dry matter; CP: crude protein; ^2^ Premix provided the following per kg of feed: vitamin A, 10,000 IU; vitamin D_3_, 3,000 IU; vit-amin E, 24 mg; vitamin K_3_, 2.1 mg; vitamin B_12_, 2 mg; riboflavin, 5.0 mg; pantothenic acid, 15 mg; niacin, 40 mg; choline chloride, 500 mg; folic acid, 0.9 mg; vitamin B_6_, 3.0 mg; biotin, 0.05 mg; Mn (from MnSO_4_·H_2_O), 70 mg; Fe (from FeSO_4_·H_2_O), 80 mg; Zn (from ZnSO_4_·H_2_O), 100 mg; Cu (from CuSO_4_·5 H_2_O), 18.8 mg; I (from KI), 0.35 mg; Se (from Na_2_SeO_3_), 0.30 mg; ^3^ The metabolizable energy and crude protein in nutrient levels were analyzed values, other nutrients were cacluated values.

**Table 2:** Growth performance of broiler chickens as affected by dietary MOA supplementation.

Item	^1^ Added Mixed Organic Acid (%)			SEM	*p*-Value		
	0	0.3	0.6		ANOVA	Linear	Quadratic
Starter (d 1–d 21)							
Initial weight (g)	48.43	48.85	48.11	0.88	0.84	0.80	0.60
Final weight (g)	596.25	644.72	616.10	16.67	0.17	0.42	0.09
ADG (g)	26.45	28.37	27.05	0.66	0.15	0.53	0.07
ADFI (g)	35.72	36.77	36.82	1.20	0.77	0.53	0.75
FCR	1.35	1.30	1.36	0.03	0.34	0.77	0.16
Grower (d 22–d 42)							
Final weight (g)	1643.9 ^b^	1826.58 ^a^	1776.17 ^a^	25.24	<0.01	<0.01	<0.01
ADG (g)	50.23 ^b^	56.23 ^a^	55.18 ^a^	1.04	<0.01	<0.01	0.02
ADFI (g)	97.06	98.69	98.19	2.59	0.90	0.76	0.74
FCR	1.94 ^a^	1.76 ^b^	1.78 ^ab^	0.04	0.03	0.03	0.09
Overall (d 1–d 42)							
Initial weight (g)	48.43	48.85	48.11	0.88	0.84	0.80	0.60
Final weight (g)	1643.9 ^b^	1826.58 ^a^	1776.17 ^a^	25.24	0.01	0.01	0.01
ADG (g)	37.99 ^b^	42.33 ^a^	41.14 ^a^	0.61	0.01	0.01	0.01
ADFI (g)	66.38	67.72	67.50	1.22	0.72	0.05	0.61
FCR	1.75 ^a^	1.60 ^b^	1.64 ^a,b^	0.03	0.03	0.05	0.04

^a,b^ In each row, means with the same letter represented no significant differences; ^1^ CON: broilers supplementing a basic diet; LMOA: broilers fed a basal diets supplemented with 0.3% (3000 mg/kg) mixed organic acids; HMOA: broilers fed a basal diets supplemented with 0.6 % (6000 mg/kg) mixed organic acids. ADG: average daily gain; ADFI: average daily feed intake; FCR: feed conversion ratio. *n* = 8.

**Table 3:** Breast and thigh muscle quality of 42 d broiler chickens as affected by dietary MOA supplementation.

Item	^1^ Added Mixed Organic Acid, %			SEM	*p*-Value		
	0	0.3	0.6		ANOVA	Linear	Quadratic
Breast muscle							
pH_45 min_	6.79	6.72	6.57	0.09	0.26	0.12	0.69
^2^ L*_45 min_	60.45	59.54	58.38	0.78	0.22	0.09	0.89
a*_45 min_	15.16	15.51	15.63	0.38	0.68	0.41	0.80
b*_45 min_	16.18	14.65	15.46	0.39	0.06	0.22	0.04
pH_24 h_	5.75 ^b^	5.80 ^a,b^	5.86 ^a^	0.02	0.02	0.01	0.84
^3^ L*_24 h_	66.32	65.38	66.70	0.76	0.48	0.73	0.25
a*_24 h_	13.05	13.50	13.90	0.28	0.15	0.06	0.93
b*_24 h_	18.13	17.74	16.56	0.67	0.27	0.13	0.64
^4^ ΔpH	1.04 ^a^	0.93 ^ab^	0.71 ^b^	0.10	0.04	0.04	0.68
Drip loss (%)	2.33	2.47	2.57	0.18	0.65	0.36	0.94
Thigh muscle							
pH_45 min_	6.62	6.63	6.64	0.05	0.96	0.79	0.99
L*_45 min_	63.63	64.67	64.21	1.39	0.87	0.77	0.67
a*_45 min_	14.86	15.97	15.60	0.48	0.30	0.30	0.24
b*_45 min_	18.92	18.79	18.94	0.69	0.98	0.98	0.87
pH_24 h_	5.83 ^b^	6.00 ^a,b^	6.07 ^a^	0.06	0.04	0.02	0.59
L*_24 h_	66.95	66.86	66.52	1.15	0.96	0.80	0.93
a*_24 h_	13.06 ^b^	13.71 ^a,b^	15.15 ^a^	0.51	0.04	0.02	0.53
b*_24 h_	20.86	20.78	19.93	0.43	0.29	0.16	0.49
ΔpH	0.79	0.63	0.57	0.08	0.21	0.09	0.70
Drip loss (%)	1.98	1.76	1.79	0.12	0.42	0.31	0.40

^a,b^ In each row, means with the same letter represented no significant differences; ^1^ CON: broilers supplementing a basic diet; LMOA: broilers fed a basal diets supplemented with 0.3% (3000 mg/kg) mixed organic acids; HMOA: broilers fed a basal diets supplemented with 0.6 % (6000 mg/kg) mixed organic acids. *n* = 8; ^2^ L*_45 min_, a*_45 min_, b*_45 min_, luminosity, redness and yellowness at 45 min; ^3^ L*_24 h_, a*_24 h_, b*_24 h_, luminosity, redness and yellowness at 24 h; ^4^ ΔpH = pH _45 min_ − pH _24 h_.

**Table 4:** Fatty acids composition in breast muscle of broiler chickens as affected by dietary MOA supplementation (g/100 g total fatty acids, DM-basis).

Item	^1^ Added Mixed Organic Acid (%)			SEM	*p*-Value		
	0	0.3	0.6		ANOVA	Linear	Quadratic
C12:0	0.06	0.04	0.05	0.01	0.15	0.22	0.11
C14:0	0.44	0.44	0.43	0.03	0.99	0.89	0.94
C15:0	0.09	0.09	0.10	0.01	0.64	0.37	1.00
C16:0	22.24 ^a^	20.89 ^b^	21.61 ^a,b^	0.30	0.08	0.22	0.05
C17:0	0.17	0.18	0.17	0.01	0.88	0.99	0.64
C18:0	10.82	9.15	10.15	0.59	0.24	0.46	0.14
C20:0	0.18	0.16	0.18	0.01	0.21	0.81	0.10
C21:0	0.81	0.71	0.85	0.06	0.42	0.68	0.23
C22:0	0.18	0.13	0.15	0.02	0.23	0.31	0.16
C23:0	0.05	0.05	0.06	0.01	0.73	0.46	0.93
C24:0	0.12	0.08	0.10	0.01	0.25	0.42	0.15
^2^ SFA	35.15 ^a^	31.93 ^b^	33.86 ^a^	0.47	0.02	0.12	0.01
C14:1	0.07	0.06	0.06	0.01	0.83	0.57	0.91
C16:1	2.42	2.51	2.39	0.27	0.95	0.95	0.76
C18:1n9c	26.61	28.68	26.97	1.44	0.60	0.87	0.35
C20:1	0.30	0.29	0.30	0.01	0.78	0.87	0.52
C22:1n9	0.13	0.09	0.12	0.02	0.38	0.66	0.20
C24:1	0.23	0.19	0.25	0.03	0.40	0.69	0.22
MUFA	29.76	31.82	30.08	1.69	0.68	0.90	0.41
C18:3n3	1.54 ^b^	2.05 ^a^	1.78 ^a,b^	0.11	0.08	0.20	0.04
C20:3n3	0.12	0.10	0.12	0.01	0.47	0.84	0.25
C20:5n3	0.44	0.30	0.35	0.04	0.15	0.20	0.11
C22:6n3	1.41	1.12	1.41	0.20	0.53	0.99	0.29
Total N-3 FA	3.50	3.57	3.67	0.19	0.82	0.56	0.95
C18:2n6c	25.17	27.89	26.75	0.70	0.12	0.18	0.09
C20:3n6	0.96	0.76	0.94	0.07	0.18	0.87	0.08
C20:4n6	5.46	4.02	4.71	0.74	0.46	0.51	0.31
Total N-6 FA	31.59	32.67	32.39	1.15	0.80	0.65	0.65
PUFA	35.10	36.25	36.06	1.31	0.81	0.63	0.70
UFA	64.85 ^b^	68.07 ^a^	66.14 ^b^	0.47	0.02	0.12	0.01
N-6/N-3	9.05	9.15	8.86	0.24	0.70	0.60	0.53
P/S	1.00 ^b^	1.14 ^a^	1.07 ^a,b^	0.03	0.06	0.16	0.04

^a,b^ In each row, means with the same letter represented no significant differences; ^1^ CON: broilers supplementing a basic diet; LMOA: broilers fed a basal diets supplemented with 0.3% (3000 mg/kg) mixed organic acids; HMOA: broilers fed a basal diets supplemented with 0.6 % (6000 mg/kg) mixed organic acids. *n* = 8; ^2^ SFA: saturated fatty acids; MUFA: monounsaturated fatty acids; Total N-3 FA: Total N-3 fatty acids; Total N-6 FA: Total N-6 fatty acids; PUFA: polyunsaturated fatty acids; UFA: unsaturated fatty acids (MUFA+PUFA); N-6/N-3: Total N-6 FA / Total N-3 FA; P/S: PUFA/SFA.

**Table 5:** Fatty acids composition in thigh muscle of broiler chickens as affected by dietary MOA supplementation (g/100 g total fatty acids, DM-basis).

Item	^1^ Added Mixed Organic Acid (%)			SEM	*p*-Value		
	0	0.3	0.6		ANOVA	Linear	Quadratic
C12:0	0.03	0.03	0.03	0.01	0.44	0.29	0.52
C14:0	0.49	0.50	0.52	0.02	0.56	0.33	0.76
C15:0	0.08 ^b^	0.10 ^a^	0.10 ^a^	0.01	0.05	0.05	0.07
C16:0	22.19	21.28	21.98	0.37	0.30	0.71	0.15
C17:0	0.15	0.16	0.16	0.01	0.73	0.62	0.57
C18:0	8.41	7.30	7.50	0.73	0.58	0.43	0.52
C20:0	0.16	0.15	0.15	0.01	0.37	0.29	0.36
C21:0	0.42	0.42	0.42	0.01	0.99	0.97	0.98
C22:0	0.16 ^a^	0.12 ^a,b^	0.10 ^b^	0.01	0.07	0.03	0.70
C23:0	0.05 ^a^	0.04 ^a,b^	0.04 ^b^	0.01	0.08	0.04	0.42
C24:0	0.11	0.10	0.06	0.01	0.11	0.05	0.55
^2^ SFA	32.26	30.23	31.04	0.69	0.22	0.28	0.17
C14:1	0.11	0.10	0.12	0.02	0.69	0.52	0.60
C16:1	4.09	3.89	4.28	0.59	0.90	0.83	0.71
C18:1n9c	31.39	31.41	30.88	1.41	0.96	0.81	0.88
C20:1	0.29	0.28	0.27	0.01	0.61	0.36	0.85
C22:1n9	0.07	0.06	0.07	0.01	0.72	0.75	0.48
C24:1	0.12	0.10	0.09	0.02	0.58	0.34	0.75
MUFA	36.08	35.85	35.72	1.84	0.99	0.90	0.98
C18:3n3	1.85 ^b^	2.25 ^a^	2.08 ^a,b^	0.09	0.08	0.14	0.05
C20:3n3	0.06	0.06	0.06	0.02	0.98	0.89	0.94
C20:5n3	0.22	0.21	0.22	0.02	0.98	0.92	0.86
C22:6n3	0.70	0.68	0.74	0.18	0.97	0.88	0.87
Total N-3 FA	2.83	3.19	3.10	0.19	0.44	0.36	0.38
C18:2n6c	25.67 ^b^	28.20 ^a^	27.10 ^a,b^	0.60	0.10	0.17	0.05
C20:3n6	0.54	0.50	0.52	0.08	0.94	0.87	0.78
C20:4n6	2.63	2.04	2.52	0.53	0.72	0.90	0.45
Total N-6 FA	28.84	30.73	30.14	1.14	0.54	0.46	0.42
PUFA	31.66	33.93	33.24	1.32	0.52	0.45	0.41
UFA	67.74	69.77	68.96	0.69	0.22	0.28	0.17
N-6/N-3	10.25	9.77	9.65	0.26	0.33	0.27	0.32
P/S	0.98 ^b^	1.12 ^a^	1.07 ^a,b^	0.03	0.09	0.14	0.04

^a,b^ In each row, means with the same letter represented no significant differences; ^1^ CON: broilers supplementing a basic diet; LMOA: broilers fed a basal diets supplemented with 0.3% (3000 mg/kg) mixed organic acids; HMOA: broilers fed a basal diets supplemented with 0.6 % (6000 mg/kg) mixed organic acids. *n* = 8; ^2^ SFA: saturated fatty acids; MUFA: monounsaturated fatty acids; Total N-3 FA: Total N-3 fatty acids; Total N-6 FA: Total N-6 fatty acids; PUFA: polyunsaturated fatty acids; UFA: unsaturated fatty acids (MUFA+PUFA); N-6/N-3: Total N-6 FA / Total N-3 FA; P/S: PUFA/SFA.

**Table 6:** Small intestine morphology of 21 d broiler chickens as affected by dietary MOA supplementation.

Item	^1^ Added Mixed Organic Acid (%)			SEM	*p*-Value		
	0	0.3	0.6		ANOVA	Linear	Quadratic
Duodenum							
Villus height (μm)	994.05	934.22	975.83	56.90	0.75	0.83	0.48
Crypt depth (μm)	94.35 ^a^	92.51 ^ab^	84.73 ^b^	2.82	0.08	0.04	0.41
^2^ V/C	10.67	10.11	11.65	0.57	0.20	0.25	0.16
Jejunum							
Villus height (μm)	682.05 ^b^	754.65 ^ab^	834.13 ^a^	26.25	0.01	0.01	0.92
Crypt depth (μm)	86.23	91.69	82.12	7.74	0.69	0.71	0.45
V/C	8.39	8.55	10.58	0.82	0.16	0.09	0.37
Ileum							
Villus height (μm)	419.09	437.00	443.32	14.1	0.48	0.25	0.74
Crypt depth (μm)	83.34 ^a^	82.22 ^a,b^	67.64 ^b^	4.76	0.08	0.04	0.28
V/C	5.18 ^b^	5.47 ^b^	6.73 ^a^	0.36	0.03	0.01	0.29

^a,b^ In each row, means with the same letter represented no significant differences; ^1^ CON: broilers supplementing a basic diet; LMOA: broilers fed a basal diets supplemented with 0.3% (3000 mg/kg) mixed organic acids; HMOA: broilers fed a basal diets supplemented with 0.6 % (6000 mg/kg) mixed organic acids. *n* = 8; ^2^ V/C: villus height/crypt depth.

**Table 7:** Small intestine morphology of 42 d broiler chickens as affected by dietary MOA supplementation.

Item	^1^ Added Mixed Organic Acid (%)			SEM	*p*-Value		
	0	0.3	0.6		ANOVA	Linear	Quadratic
Duodenum							
Villus height (μm)	962.07 ^b^	1203.81 ^a^	1189.44 ^a^	50.67	0.01	0.01	0.06
Crypt depth (μm)	80.23 ^b^	87.45 ^a,b^	98.13 ^a^	4.88	0.07	0.03	0.78
^2^ V/C	12.51	13.97	12.34	0.82	0.34	0.88	0.15
Jejunum							
Villus height (μm)	864.90	1013.80	1048.40	70.76	0.21	0.10	0.53
Crypt depth (μm)	87.40 ^b^	107.30 ^a^	101.56 ^a,b^	4.80	0.05	0.07	0.06
V/C	10.36	9.51	10.64	1.02	0.73	0.85	0.45
Ileum							
Villus height (μm)	523.80 ^b^	703.83 ^a^	658.40 ^a,b^	53.28	0.05	0.10	0.11
Crypt depth (μm)	78.42	89.06	83.71	6.86	0.57	0.60	0.36
V/C	6.81	8.02	8.05	0.42	0.10	0.06	0.28

^a,b^ In each row, means with the same letter represented no significant differences; ^1^ CON: broilers supplementing a basic diet; LMOA: broilers fed a basal diets supplemented with 0.3% (3000 mg/kg) mixed organic acids; HMOA: broilers fed a basal diets supplemented with 0.6 % (6000 mg/kg) mixed organic acids. *n* = 8; ^2^ V/C: villus height/crypt depth.

**Table 8:** Volatile fatty acids in cecum content of 21 d and 42 d broiler chickens as affected by dietary MOA supplementation (mg/g).

Item	^1^ Added Mixed Organic Acid (%)			SEM	*p*-value		
	0	0.3	0.6		ANOVA	Linear	Quadratic
21 d							
Lactic acid	74.13	122.04	112.95	18.34	0.19	0.17	0.23
Acetic acid	3143	2831	3111	266.36	0.67	0.93	0.39
Propionic acid	300.38	281.87	251.23	44.22	0.74	0.45	0.91
Formic acid	6.64 ^b^	15.46 ^a^	16.17 ^a^	2.00	0.01	0.01	0.13
Isobutyric acid	45.45	43.69	39.62	6.58	0.82	0.54	0.89
Butyric acid	473.90	569.20	465.40	72.89	0.55	0.94	0.29
Isovaleric acid	22.20	14.36	21.93	6.55	0.64	0.98	0.36
Valeric acid	19.80	28.01	18.08	4.68	0.32	0.80	0.14
Total volatile fatty acids	4086	3906	4037	352.60	0.93	0.92	0.73
42 d							
Lactic acid	101.43	77.35	42.74	18.88	0.14	0.05	0.82
Acetic acid	4084 ^b^	4897 ^a^	3906 ^b^	207.26	0.02	0.56	0.01
Propionic acid	724.66 ^b^	1050.01 ^a^	688.69 ^b^	43.03	0.01	0.57	0.00
Formic acid	13.31 ^a,b^	13.88 ^a^	7.31 ^b^	1.97	0.07	0.06	0.17
Isobutyric acid	68.44 ^a^	125.25 ^a^	80.48 ^b^	9.56	0.01	0.39	0.01
Butyric acid	1100 ^b^	1783 ^a^	1079 ^b^	86.64	0.01	0.86	0.01
Isovaleric acid	64.09	57.94	53.95	4.80	0.36	0.16	0.86
Valeric acid	39.62	53.23	37.17	8.72	0.41	0.85	0.20
Total volatile fatty acids	6196 ^b^	8058 ^a^	5895 ^b^	310.40	0.01	0.51	0.01

^a,b^ In each row, means with the same letter represented no significant differences; ^1^ CON: broilers supplementing a basic diet; LMOA: broilers fed a basal diets supplemented with 0.3 % (3000 mg/kg) mixed organic acids; HMOA: broilers fed a basal diets supplemented with 0.6 % (6000 mg/kg) mixed organic acids. *n* = 8.

## Data Availability

The original contributions generated for this study are included in the article, further inquiries can be directed to the corresponding author.
